# An integrated machine learning-based prognostic model in head and neck cancer using the systemic inflammatory response index and correlations with patient reported financial toxicity

**DOI:** 10.21203/rs.3.rs-6529613/v1

**Published:** 2025-05-07

**Authors:** Anurag K. Singh, Sung Jun Ma, Dukagjin Blakaj, Simeng Zhu, Neil D. Almeida, Andrew Koempel, Guangwei Yuan, Grace Wang, Kimberly Wooten, Vishal Gupta, Ryan McSpadden, Moni A. Kuriakose, Michael R. Markiewicz, Song Yao, Wesley L. Hicks, Mukund Seshadri, Elizabeth A. Repasky, Elizabeth G. Bouchard, Mark K. Farrugia, Han Yu

**Affiliations:** a Department of Radiation Medicine Roswell Park Comprehensive Cancer Center Elm and Carlton Streets Buffalo, NY 14203. USA.; b Jacobs School of Medicine and Biomedical Sciences University at Buffalo, The State University of New York 955 Main Street Buffalo, NY 14203. USA.; d University at Buffalo, The State University of New York 12 Capen Hall Buffalo, NY 14260. USA.; e Department of Head and Neck Surgery Roswell Park Comprehensive Cancer Center Elm and Carlton Streets Buffalo, NY 14203. USA.; f Department of Biostatistics and Bioinformatics Roswell Park Comprehensive Cancer Center Elm and Carlton Streets Buffalo, NY 14203. USA.; g Department of Oral and Maxillofacial Surgery School of Dental Medicine University at Buffalo, The State University of New York 3435 Main Street Buffalo, NY 14214. USA.; h Department of Neurosurgery Department of Surgery Jacobs School of Medicine and Biomedical Sciences University at Buffalo, The State University of New York 955 Main Street Buffalo, NY 14203. USA.; i Department of Oral Oncology Roswell Park Comprehensive Cancer Center Elm and Carlton Streets Buffalo, NY 14203. USA.; j Department of Cancer Prevention and Control Roswell Park Comprehensive Cancer Center Elm and Carlton Streets Buffalo, NY 14203. USA.; k Department of Immunology Roswell Park Comprehensive Cancer Center Elm and Carlton Streets Buffalo, NY 14203. USA.; l Department of Radiation Oncology, The Arthur G. James Cancer Hospital and Richard J. Solove Research Institute, The Ohio State University Comprehensive Cancer Center, 460 West 10th Avenue, Columbus, OH, 43210, USA

**Keywords:** Inflammation, neutrophil, lymphocyte, monocyte, squamous cell carcinoma

## Abstract

**Objective:**

To investigate the prognostic utility of systemic inflammatory response index (SIRI) as a biological readout of stress associated immune modulation in head and neck cancer patients who underwent radiation therapy.

**Methods:**

Random survival forest machine learning was used to model survival in 568 head and neck cancer patients. SIRI was calculated via pre-treatment bloodwork. Model validation was performed in an external cohort of 345 patients. Baseline financial toxicity (FT) and SIRI were studied in 638 patients.

**Results:**

Incorporation of SIRI (with performance status and smoking history) into a machine learning model identified three risk-groups that significantly stratified overall survival (p<0.0001,) and these findings were validated in the external validation cohort (p<0.001.) Increasing levels of FT were significantly associated with increasing SIRI levels. (p=0.001.)

**Conclusions and Relevance:**

An integrated machine learning model using clinical features was successfully developed and externally validated. SIRI was significantly associated with increasing FT. Our findings highlight the potential utility of SIRI as a biological marker of FT in head and neck cancer patients.

The second century Roman physician Galen noted that cancer was more likely to afflict the melancholic.^1^ Emotional distress activates both the hypothalamic-pituitary-adrenal axis and sympathetic nervous system leading to release of stress hormones including cortisol and norepinephrine.^2,3^ To this point, a recent publication in lung cancer patients showed that emotional distress, characterized by symptoms of depression or anxiety, significantly impacts response to immune checkpoint inhibitors and is associated with elevated cortisol levels.^4^ Stress hormones alter neutrophil counts, half-life, and function.^5,6^ Similarly, stress hormones regulate monocyte migration and activity.^7–10^ Conversely, stress hormones can reduce lymphocyte counts by sequestering lymphocytes in secondary lymphoid tissues.^11,12^

Systemic inflammation promotes cancer progression,^13^ which stimulates the bone marrow to produce more neutrophils.^14–16^ Increased neutrophils then release cytokines that promote angiogenesis and cancer metastasis.^17,18^ As a result, elevated neutrophil counts have been associated with worse survival outcomes in multiple cancer types including head and neck cancer (HNC).^19^ The neutrophil to lymphocyte ratio has also been shown to be a marker of HNC survival.^20^ Similarly, high monocyte values in HNC are associated with worse survival.^21^

Since there is significant diurnal variation in the systemic cortisol level (cortisol tends to be high upon waking, peaks shortly thereafter, drops rapidly then stabilizes, with a nadir at sleep), single daily cortisol levels may not be reflective of the total impact of cortisol on inflammation.^22^ Therefore, combining the longer-lived effect of stress hormones on increasing neutrophils and monocytes while decreasing lymphocytes in the peripheral blood may be a more stable marker of stress. Such changes in the peripheral blood would produce elevations in the previously defined systemic inflammatory or inflammation response index (SIRI), calculated as [(neutrophils × monocytes)/lymphocytes]. Elevated SIRI has been implicated as prognostic factor for survival in a variety of malignancies^23–27^ including HNC.^28–32^

Standard of care treatment options for advanced HNC are broad and include many possible combinations of systemic therapy, radiation, and surgery.^33^ Treatment related toxicities can be severe^34^ and frequently life threatening.^35^ Toxicities increase with increasing duration and number of modalities of therapy.^36^ Better prognostication of survival outcomes is therefore needed to optimize treatment selection to better match expected outcomes and toxicities.

Previously, we developed a prediction model using machine learning approach for HNC patients’ survival based on 13 demographic and clinical predictors plus a host factor incorporating 12 variables available from a pre-treatment complete blood count thought to reflect baseline inflammation. In this prior analysis we noted that neutrophil percentages were negatively correlated with the lymphocyte percentage. This model had a high discriminative power (hazard ratio of 7.41, *p*< 0.0001).^37^ However, the large number of variables in that model made clinical implementation challenging.

The prior machine learning model did not have any variables related to emotional distress characterized by depression or anxiety. Symptoms of depression, anxiety and cancer related worry are highly correlated with financial toxicity (FT).^38^ FT describes the psychosocial, behavioral, and material effects of subjective financial stress experienced by patients resulting from objective economic hardship, such as significant out-of-pocket medical expenses combined with disruptions in employment.^39–41^ Nearly half of all cancer patients experience FT.^39,42^ Most HNC patients require multimodality therapy and thus have a higher risk for FT.^43,44^

Our recent match-paired study revealed that high FT at baseline is associated with significantly worse overall and cancer specific survival in HNC.^45^ However, the mechanism by which FT caused worse survival remains elusive. A clinical readout of FT would provide mechanistic insight and assist in evaluating the effectiveness of interventions to mitigate FT.

In this context, we hypothesized that SIRI may serve as a valuable clinical marker of outcomes and correlate with FT related emotional distress in HNC. To test this hypothesis, we developed and validated a machine learning model that integrated clinical features including SIRI in head and neck cancer patients undergoing radiation therapy. Specifically, we examined if: 1) inclusion of SIRI in a machine learning model would produce a clinically usable prognostic model for HNC survival outcomes and 2) emotional distress as measured by degree of FT would be correlated with SIRI values.

## Results

Patient characteristics are shown in Table 1. Median follow up was 33.1 months (interquartile range 13–71.3 months). Our previous work identified Karnofsky Performance Status (KPS), body mass index (BMI), smoking status, and a composite host factor score as the top predictors of overall survival in head and neck cancer patients.^33^ In this study, we explore whether substituting the more complex host factor score with the SIRI allows for a simplified model that maintains comparable predictive performance using only the other top predictors.

**Figure 1.**
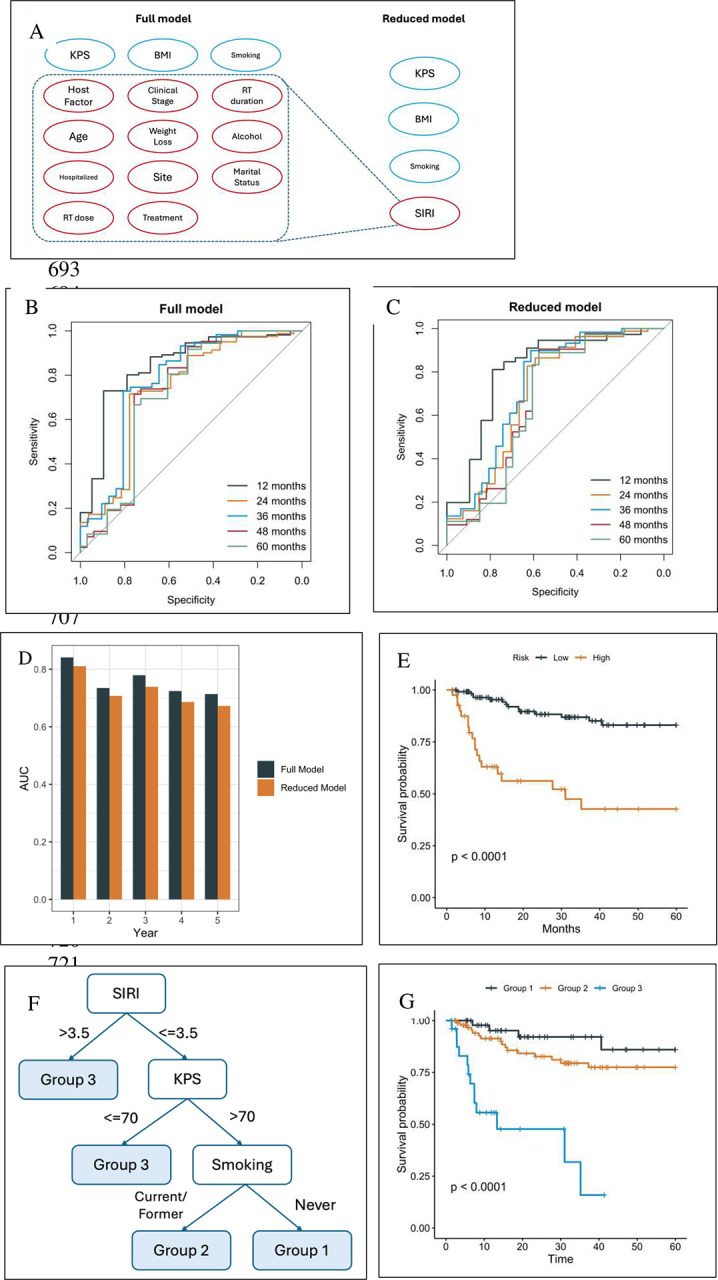
(A) In the current, reduced model, SIRI replaced several predictors from the prior, full model. (B) Receiver operating characteristic curves (ROCs) of the full model. (C) ROCs of the 4-feature model. (D) Comparison of the area under the curve (AUCs) at months 12–60. (E) The Kaplan-Meier curves based on the risk stratification by the reduced model. (F) The decision tree of the parsimonious model partitions the cohort into four groups, where two high-risk groups were merged to Group 3. (G) The Kaplan-Meier curves of the three groups in the test set.

Figure 1A shows the variables utilized in the prior and current model with only 4 variables (SIRI, Karnofsky Performance Status (KPS), body mass index (BMI), and smoking status) in predicting overall survival. As shown in Figure 1B–C, the ROC curves for the prior (full) and current (reduced) model indicate that both models perform well in terms of discriminative power. Notably, the difference in AUCs between the two models remains small, with the maximum difference consistently below 0.05, as illustrated in Figure 1D. This suggests that the reduced model retains a comparable level of predictive accuracy, making it a viable alternative for situations where model complexity and ease of use are critical considerations.

Since our primary goal is to identify patients at the highest risk, the test cohort was further stratified based on the 75^th^ percentile of the predicted risk scores derived from the simplified model. Figure 1E illustrates that, patients in the high-risk group, as defined by this threshold, experience significantly worse overall survival compared to those in the low-risk group (hazard ratio 5.1, 95% confidence interval p<0.0001). Therefore, the reduced model based on the four selected variables is sufficient to attain comparable performance versus the full model.

Random survival forests (RSF) models are effective but complex and unintuitive, making them difficult to implement in clinical practice. In contrast, decision trees are simple, intuitive and naturally aligned with clinical decision-making. However, they are prone to high instability during model training. To construct a more robust decision tree, prior to the tree growing process, we identified the potentially optimal cutoffs of: KPS =70, BMI = 25 and 30, and SIRI = 3.5. Stratification by these 4 variables created 6 risk groups. However, the survival curves of these groups clustered effectively as 3 groups (Figure S2.) Therefore, the reduced model was adjusted, and BMI was removed as a variable to create a further simplified, parsimonious model. This parsimonious model decision tree partitions the cohort into 3 groups (Figure 1F.) The C-index of the models were: 0.758 (RSF full), 0.725 (RSF reduced), 0.709 (decision tree reduced), and 0.702 (decision tree parsimonious). The parsimonious model was chosen for further study. Analysis of the internal validation cohort shows significant differences in overall survival among the 3 risk groups (Figure 1G, p<0.0001.)

**Figure 2.**
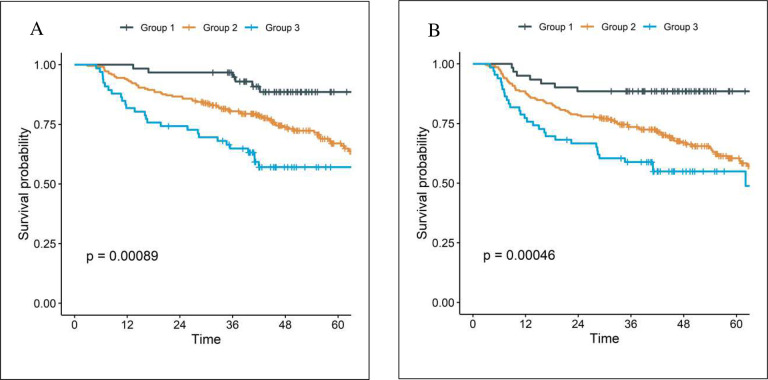
The Kaplan-Meier curves of (A) overall survival, (B) progression free survival from the External Validation Cohort.

The external validation cohort of the parsimonious, 3 variable model treated at The Ohio State University consisted of 345 patients with 82.3% men and median age 61 years [interquartile range 55–68]). Most patients were either former [38.8%] or current [39.1%] smokers with oropharyngeal cancer [63.8%] who received definitive chemoradiation [81.7%]. Median follow up was 49.7 months (interquartile range 41.3–63.3). Kaplan-Meier and cumulative incidence plots are shown in Figure 2. There was a statistically significant difference among Groups for OS (5-year OS: 88.6% for Group 1, 67.0% for Group 2, 57.1% for Group 3; p<0.001) and PFS (5-year PFS: 88.5% for Group 1, 60.4% for Group 2, 54.9% for Group 3; p<0.001). With 3 comparisons (Groups 1 vs 2, 1 vs 3, and 2 vs 3) and a Holm-Bonferroni correction, all comparisons remained statistically significant for OS (Groups 2 vs 1: hazards ratio [HR] 2.56, 95% confidence interval [CI] 1.24–5.32, p=0.011; Groups 3 vs 1: HR 4.10, 95% CI 1.87–9.01, p<0.001; Groups 3 vs 2: HR 1.60, 95% CI 1.03–2.47, p=0.035). For PFS, only the comparison between groups 2 and 3 was not significant (Groups 2 vs 1: HR 3.16, 95% CI 1.53–6.52, p=0.002; Groups 3 vs 1: HR 4.38, 95% CI 2.00–9.56, p<0.001; Groups 3 vs 2: HR 1.38, 95% CI 0.91–2.10, p=0.13).

**Figure 3:**
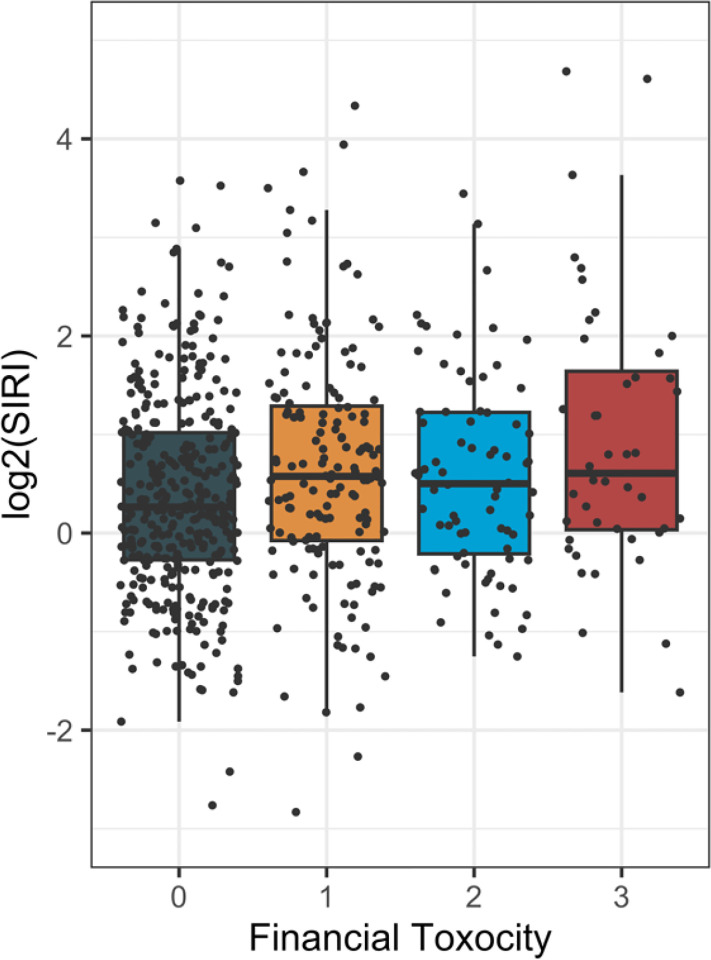
Distribution of log_2_(SIRI) among FT groupings, p=0.001.

To study the association at baseline, the distribution of log_2_(SIRI) by degree of FT was plotted as shown in Figure 3. SIRI increased significantly for every increase in FT (p=0.001.) To account for the potential confounding factors, a multiple linear regression analysis was performed where the log_2_(SIRI) was treated as the outcome variable. Table 2 shows the positive association between SIRI and financial toxicity (p=0.017.) In addition, the older (p<0.002) and white patients (p<0.001) also have significantly higher SIRI scores on average. The disease site of salivary gland is also associated with lower SIRI compared with the larynx (p=0.016). Meanwhile, patients who received RT only (p=0.010) or surgery/RT (p<0.001) had higher SIRI versus those received CCRT.

## Discussion

Here we present the prognostic utility of a machine learning model based on novel 3-feature signature using routinely available pre-treatment clinical variables (SIRI, KPS, and smoking history) along with patient reported FT in head and neck cancer. This analysis produced 2 extremely novel and important findings. First, aided by machine learning, a parsimonious model with only 3 (SIRI, performance status, and smoking history) variables was developed and validated as a significant predictor of overall and progression free survival for HNC. Second, demonstration of a significant association of financial toxicity and SIRI in any population is a novel finding (p=0.001.) Better prognostication of survival outcomes may allow clinicians and HNC patients to make more informed choices about treatment intensity to better match expected outcomes and toxicities.

Valero et al. studied 23 clinical and genomic variables that could predict PFS after immunotherapy in HNC. Concordant with our findings, in their machine learning model smoking, SIRI, and performance status were the first, second, and sixth most important out of 23 total variables including several genomic markers,.^46^

Machine learning models with a great many variables can closely approximate survival, but due to complexity, are virtually impossible to deploy in the clinic. The full and reduced RSF models had a C-index of 0.758 and 0.725, respectively. The reduced and parsimonious decision tree models had a lower C-index of 0.709 to 0.702. This relatively minor penalty in C-Index was certainly worth the increased simplicity which will considerably improve the applicability in clinical settings of the parsimonious decision tree model.

Proof of the independence of the machine learning process comes from grouping together of current and former smokers in the model. We previously found that former-smokers are distinct from current smokers and have similar survival to non-smokers.^47^ Additionally, though eventually dropped from the parsimonious model, the selected cut-points for BMI are around 25 and 30; these are the standard thresholds for being overweight and obese. Interestingly, these are not the thresholds we found when we previously studied BMI^48^ as an independent variable. The concordance of our machine learning defined cut-points and standard threshold for BMI and discordance with our own previously published thresholds demonstrates the validity of our method and independence of the machine learning process from external influence.

The current analysis is the first to show that SIRI significantly increases with increasing financial toxicity (Figure 3.) The significant association of SIRI and FT was maintained after multiple linear regression. An abstract with 65 prostate cancer patients found a significant correlation between baseline tumor necrosis factor alpha and FT.^49^ Stress hormones are generally thought to inhibit tumor necrosis factor alpha, but the effects of chronic stress may be contradictory, leaving the overall effect unclear.^50^

On multiple linear regression analysis, in addition to FT, older age (p<0.002), white race (p<0.001), and treatment variables (RT only (p=0.010), surgery/RT (p<0.001) versus CCRT) were associated with significantly higher SIRI scores on average. Increased SIRI with demographic variables is consistent with several studies. Rundle et al. in a study of patients with benign finding on prostate biopsy, found that the ratios which combine to form SIRI were significantly elevated in white race control patients compared to those who went on to develop prostate cancer; case/control differences were not seen in black patients.^51^ Older patients tend to have increased inflammation^52^ characterized by increasing neutrophil counts and decreasing lymphocyte counts which would produce a higher SIRI. In contrast to our findings, in a non-cancer population, other studies have found that females have higher neutrophil to lymphocyte ratios than men.^53^ This finding is decreased by the effect of ageing.^54^ If eventually validated, a biomarker of FT may illuminate, and assist in evaluating, future mitigation strategies.

Development and validation of the parsimonious 3 variable decision tree has multiple implications for improved treatment selection in HNC. Current guidelines offer a multitude of standard of care methods to both escalate (addition of immunotherapy, induction chemotherapy, surgery, novel therapies) and de-escalate (reduced radiation dose or duration) the intensity and duration of curative therapy.^33^ However, perhaps due to improper risk stratification, none of these alternatives have shown an overall survival benefit. Moreover, future studies may attempt to modulate SIRI pre-treatment to improve outcome.

Our retrospective study has inherent limitations. Regarding the machine learning model, we attempted to ameliorate these limitations by using an external validation cohort. The simplified model defined 3 groups with distinct survivals. It is known that the combination of human papilloma virus (HPV) status of the tumor and 10 or fewer pack years of smoking history defines 3 groups with distinct survival in HNC of the oropharynx.^55^ Of note, oropharynx patients in this cohort similarly segregate into 3 groups with distinct survival.^47^ The machine learning model had access to these variables but did not use them in either the full or reduced models. The benefits of the presented model are: 1) the model applies to all HNC sites as shown in the external validation cohort and 2) SIRI may be a novel readout of interventions aimed at mitigating FT in HNC patients.

No external validation cohort to test the association between FT and SIRI was available for analysis. External validation of these findings is needed. We recently showed that HNC patients with high FT at baseline had both significantly worse overall and cancer specific survival.^45^ Until now, much of the literature has focused on changed patient decision making or access to care to explain altered survival with FT.^39,56^ If SIRI is validated as a biomarker of FT, it will illuminate a biological mechanism by which a emotional distress, such as from FT, impacts survival.

The mechanism by which FT induced emotional distress may increase SIRI cannot be addressed by our analysis. Unfortunately, samples to measure pretreatment stress hormones, such as cortisol, were not available.

## Conclusion

A machine learning model including only 3 readily available pretreatment factors (SIRI, performance status, and smoking history) is validated and defines 3 groups with distinct survival outcomes for head and neck cancer. Patients with higher FT had significantly higher SIRI. The significant positive association between SIRI and FT was retained after accounting for potential confounding factors.

## Methods

Our study was performed under a protocol (EDR 103707) approved by Roswell Park Comprehensive Cancer Center institutional review board. The Strengthening the Reporting of Observational Studies in Epidemiology (STROBE) reporting guideline was reviewed, and our study follows the guideline.

Our retrospective database included all patients with primary HNC who underwent curative-intent definitive or post-operative radiation therapy at the Roswell Park Comprehensive Cancer Center between January 2013 and April 2024. Patients were excluded if they had radiation therapy alone, were diagnosed with metastatic cancer, or had unknown QOL.

We included 636 head and neck cancer patients who received radiotherapy, with available data on their pre-treatment SIRI and pre-treatment financial toxicity survey responses. The machine learning cohort included 568 of these patients. All patients received definitive radiation dose as appropriate based on the NCCN guideline (63–65.25 Gy in 28–29 fractions for T1-T2 glottic laryngeal cancer; 69.96 Gy in 33 fractions or 70 Gy in 35 fractions for all others per treating physician’s discretion).^33^ Details were previously published.^57,58^ The survey question from the European Organization for Research and Treatment of Cancer [EORTC] QLQ30 instrument used to assess financial toxicity was: “Has your physical condition or medical treatment caused you financial difficulties?” Respondents rated their experience on a 4-point Likert scale with 0 (not at all,) 1 (a little,) 2 (quite a bit), and 3 (very much). Responses of 0–1 were categorized as FT-Low, and 2–3 were categorized as FT-High. The baseline survey, encompassing responses from 30 days before to 7 days after the start of radiotherapy, was used in the analysis. Patient characteristics were summarized using frequencies and relative frequencies and compared between financial toxicity groups using Fisher’s exact test. Age was summarized as the median with interquartile range and compared between groups using the Wilcoxon rank-sum test. Additionally, the association between SIRI scores and financial toxicity responses (0–3) was assessed using the Jonckheere’s trend test. To further examine the relationship between financial toxicity and SIRI, multiple linear regression analysis was performed by modeling log2(SIRI) as a function of financial toxicity group and other potential confounding variables. All p-values were two-sided and those less than 0.05 were deemed statistically significant. All statistical analyses were performed using R (version 4.0.3, R Project for Statistical Computing, Vienna, Austria).

### Machine Learning Models

The Random Survival Forests (RSFs) are an extension of RFs designed for analyzing time-to-event data, utilizing tree-based ensemble machine learning methods. In comparison to linear models, the RF model generally provides better prediction performance due to its ability to handle nonlinear relationships and complex interactions among predictors. For a new patient, the RSF estimates the survival probability at any given time point (survival function) following treatment, along with the cumulative hazard function. Variable importance is employed to assess the contribution of each independent variable to the model’s predictions. In this work, we evaluated two models: Model 1, a more complex, previously developed (full) model^37^ and Model 2, a simplified (reduced) model. Model 1 contained 13 variables, and a “host factor score” calculated by principal component analysis of the pre-treatment complete blood count. Model 2 took the top 4 features of full model (KPS, host factor score, BMI, smoking) but replaced the host factor score with SIRI. The analyses were conducted using R 4.3.2 and R packages randomForestSRC,^59^ with 1000 trees and default settings.

### Performance Metrics

The model’s prediction performance for overall survival (OS) was assessed using the concordance index (C-index). The C-index is an extension of the area under the curve (AUC) that accounts for censored data. It is calculated as the proportion of concordant pairs out of the total evaluable pairs. A pair is considered concordant if the subject with the higher predicted survival probability also has a longer survival time. We also assessed the model’s ability to predict patient survival at 1 to 5 years, using time-dependent receiver operating characteristic (ROC) curves.

### Modeling Strategy

We primarily followed the modeling strategy as in Yu et al.^37^ Specifically, before any steps of model training, the cohort was randomly split into a training/validation set with 70% of subjects and a test set with 30% of subjects. Missing values were imputed using random forest imputation, which was performed without involving the outcome variables and strictly within the training, validation and test cohorts. Model 1 uses a standard principal component analysis (PCA) for dimension reduction in the standardized host factors. The PCA was performed only within the training/validation set. For details, please see.^37^ No test data set was used in any model training steps, including pre-processing or unsupervised learning by PCA. As the RSF requires minimal tuning of hyperparameters, we used the default settings with 1000 trees.

### Model Interpretation

The permutation variable importance (VIMP) was utilized to evaluate the impact of each independent variable on the model’s predictions. VIMP quantifies the drop in prediction performance (C-index) of the forest ensemble when a variable is randomly shuffled. A high positive VIMP value signifies that permuting this variable notably diminishes the model’s accuracy, highlighting it as a potentially influential predictor.

### Construction of a Robust Decision Tree

While the RSF model is effective, it is often regarded as a ‘black box,’ making it challenging to interpret and implement in practice. In contrast, decision trees naturally align with clinical decision-making processes and are highly user-friendly in clinical settings. However, they are prone to high instability during model training. To construct a more robust decision tree, we developed a customized approach by converting the continuous variables into categorical variables using potentially optimal cutoffs. To identify the cutoffs, we took advantage of the tree growing process in an RSF, where each decision rule was selected by maximizing the target statistic given the existing partition of the sample space. While this approach is greedy in nature, on average the cutoffs should be centered around the optimal ones while conditional on other predictors. Under this assumption, all splitting rules involving the given continuous predictor were extracted from the RSF model. The distribution of the corresponding cutoffs was then estimated using a kernel density estimator. Next, the estimated density was compared against the distribution of the variable estimated using the training data. The cutoffs were selected as local maxima in the difference between the two densities. The cutoffs that resulted in minor groups with less than a proportion of 10% were excluded. After converting the continuous predictors into categorical variables, a decision tree was constructed using the standard approach implemented in R randomForestSRC package.^60^ The maximal depth was set to 3 to avoid overly complicated rules, and the node size was set to 40.

### Statistical Analysis

For risk stratification, the survival curves were estimated using Kaplan–Meier product limit estimators and compared using log-rank tests. The hazard ratios (HRs) were estimated based on Cox proportional hazards models and the 95% confidence intervals (CIs) were reported.

### Methods for External Validation

This validation was performed after obtaining the Institutional Review Board approval at the Ohio State University Comprehensive Cancer Center (protocol 2024C0084). Its institutional database was queried for patients with non-metastatic head and neck cancer who received definitive radiation or chemoradiation between November 2012 and July 2020 with baseline SIRI available. All patients received definitive radiation dose as appropriate based on the NCCN guideline (63–65.25 Gy in 28–29 fractions for T1-T2 glottic laryngeal cancer; 66 Gy in 30 fractions for select early-stage oropharyngeal cancer; 69.96 Gy in 33 fractions or 70 Gy in 35 fractions for all others per treating physician’s discretion).^33^ Aside from T1-T2 glottic laryngeal cancer that was treated with 3D conformal radiation therapy, all others received intensity modulated radiation therapy. Those with unknown smoking status, performance status, or SIRI at baseline were excluded for analysis.

Clinical variables of interest were extracted, including age, gender, race, smoking status, primary disease site, BMI, Eastern Cooperative Oncology Group (ECOG) performance status, p16 status, tumor staging based on the American Joint Committee on Cancer (AJCC) 7th edition, treatment types, chemotherapy, and SIRI. BMI is stratified by underweight (<18.5), normal (18.5–24.9), overweight (25–29.9), or obese (≥30). ECOG performance status was adjusted to follow the Karnofsky performance status scale.

The primary endpoint was overall survival (OS), defined as the time interval from diagnosis to death from any cause or last follow up. Another endpoint included progression-free survival (PFS). PFS was defined as the time interval from diagnosis to death from any cause, tumor progression, or last follow up.

### Statistical Analysis for External Validation

Baseline characteristics were summarized using descriptive statistics. The validation of the model was performed after applying the cutoffs for the variables previously identified and stratifying the patient cohort into corresponding Groups. Survival outcomes were analyzed using the Kaplan-Meier plot, log-rank test, and univariable Cox proportional hazards regression stratified by Groups. Holm-Bonferroni correction was used for multiple comparison when comparing among different Groups. All P values were 2-sided and those less than 0.05 were considered statistically significant. The validation analysis was performed using R version 4.3.2 (R Group for Statistical Computing).

## Supplementary Material

1

## Figures and Tables

**Table 1:** Patient characteristics

	Roswell Park Machine Learning Cohort			The Ohio State External Validation Cohort			Roswell Park Financial Toxicity Cohort		
**N**	568			345			636		
	N or Median	% or IQR		N or Median	% or IQR		N or Median	% or IQR	
**Groups**									
1				61	17.7				
2				218	63.2				
3				66	19.1				
									
**SIRI**	1.34	(0.88, 2.19)		1.96	1.28–3.14		1.32	0.86–2.26	
									
**Age**	61	54–67		61	55–68		61	55–68	
									
**Gender**									
Female	104	18		61	17.7		148	23%	
Male	464	82		284	82.3		488	77%	
									
**Race**									
Caucasian	535	94%		306	88.7		566	93%	
Other	33	6%		39	11.3		43	7%	
Unknown							27		
									
**Smoking History**									
Never	174	31		76	22.0		171	27%	
Former	306	54		134	38.8		371	58%	
Current	88	15		135	39.1		94	94%	
									
**Site**									
Oropharynx	543	96%		220	63.8		358	57%	
Larynx	13	2.3%		94	27.2		120	19%	
Other	3	0.6%		31	9.0		155	24%	
Unknown							3		
									
**Body Mass Index**									
Normal	124	22%		96	27.8		166	0.26	
Underweight	11	1.9%		12	3.5		24	4%	
Overweight	221	39%		112	32.5		212	34%	
Obese	201	35%		124	35.9		230	36%	
Not available	11	1.9%		1	0.3		4		
									
**Karnofsky Performance Status**									
100	151	27%		204	59.0		142	22%	
80–90	363	64%		141	41.0		396	62%	
70–40	54	9%					95	15%	
<40							1	0.2%	
Not available							2		
									
									
**p16 Status**									
Negative	75	13%		135	39.1		120	29%	
Positive	388	68%		210	60.9		301	71.5%	
unknown	118	18%					215		
									
**Tumor Stage**									
1–2	337	59%		239	69.3		308	49%	
3–4	229	40%		106	30.7		322	51%	
Unknown	2	0.4%					6		
									
**Nodal Stage**									
0–1	210	37%		153	44.3		295	0.47	
2–3	353	62%		192	55.7		355	0.53	
Unknown	5	0.9%					6		
									
**Treatment type**									
Definitive chemoradiation	427	75.2%		282	81.7		415	65%	
Radiation alone	30	5.3%		63	18.3		19	3%	
Induction Chemotherapy, then definitive chemoradiation	51	9%					40	6.3%	
Surgery then Adjuvant (Chemo) Radiation	60	10.5%					46	7.2%	
Other							116	18.2%	
									
**Chemotherapy**									
No or other chemo	139	24.5%		193	55.9		160	25%	
Cisplatin	429	75.5%		152	44.1		476	75%	

**Table 2:** Result of multiple linear regression analysis for associations with SIRI.

Characteristic	Beta	95% CI^1^	p-value
FT			
High	—	—	
Low	−0.30	−0.55, −0.05	0.017
Age			
<=60	—	—	
>60	0.31	0.12, 0.51	0.002
Sex			
Female	—	—	
Male	0.19	−0.04, 0.41	0.11
Race			
African American	—	—	
American Indian	0.65	−0.38, 1.7	0.2
Asian	0.05	−1.3, 1.4	>0.9
White	0.84	0.42, 1.3	<0.001
Clinical Stage			
1	—	—	
2	−0.27	−0.66, 0.12	0.2
3	−0.16	−0.52, 0.19	0.4
4	−0.07	−0.39, 0.24	0.6
Site			
Larynx	—	—	
Lateral_neck	−0.30	−0.71, 0.12	0.2
Lip_oral_cavity	0.18	−0.22, 0.58	0.4
Nasal_cavity_paranasal_sinuses	−0.23	−0.97, 0.50	0.5
Other	−0.38	−1.5, 0.79	0.5
Pharynx	0.09	−0.18, 0.35	0.5
Salivary_glands	−0.90	−1.6, −0.17	0.016
Treatment			
CCRT	—	—	
ICT_CCRT	−0.08	−0.48, 0.32	0.7
Other	0.28	−2.0, 2.5	0.8
RT_only	0.72	0.17, 1.3	0.010
Surg_CCRT	−0.19	−0.50, 0.12	0.2
Surg_RT	0.82	0.37, 1.3	<0.001

1 CI = Confidence Interval

## Data Availability

The data underlying this article cannot be shared publicly for the privacy of individuals that participated in the study. The data are available from the corresponding author upon reasonable request.
