# Supplementary material for: An integrated machine learning-based prognostic model in head and neck cancer using the systemic inflammatory response index and correlations with patient reported financial toxicity
Source: Res Sq. 2025 May 7:rs.3.rs-6529613. Preprint. [Version 1] doi: 10.21203/rs.3.rs-6529613/v1 (PMC12083689; doi:10.21203/rs.3.rs-6529613/v1)
Supplement: 1 [file NIHPPrs6529613v1-supplement-1.pdf]

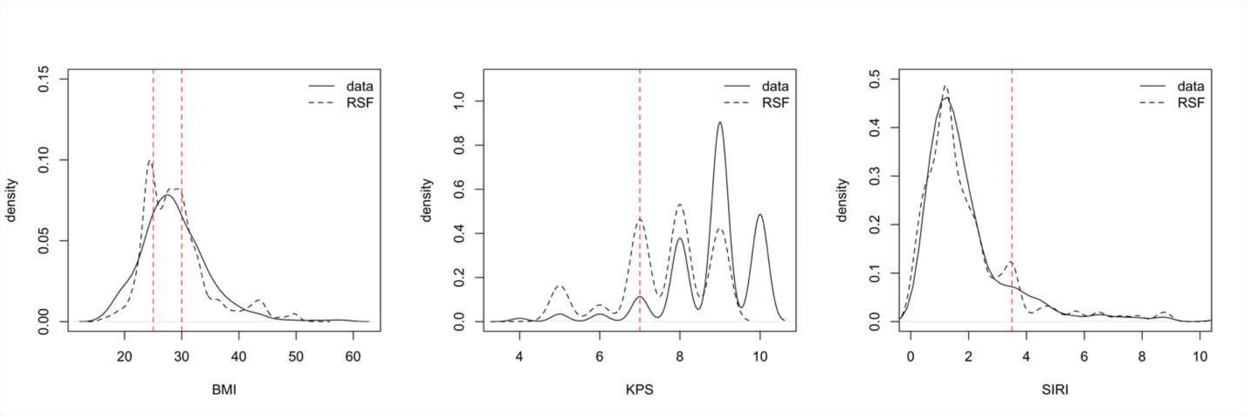

Supplemental Figure 1. Cutoff selection of three continuous variables. The distribution of the RSF cutoffs was estimated using a kernel density estimator (dashed). The estimated density was compared against the distribution of the variable estimated using the training data (solid). The cutoffs (red) were selected as local maxima in the difference between the two densities. The extreme cutoffs that would result in minor groups with less than a proportion of 10% were excluded.

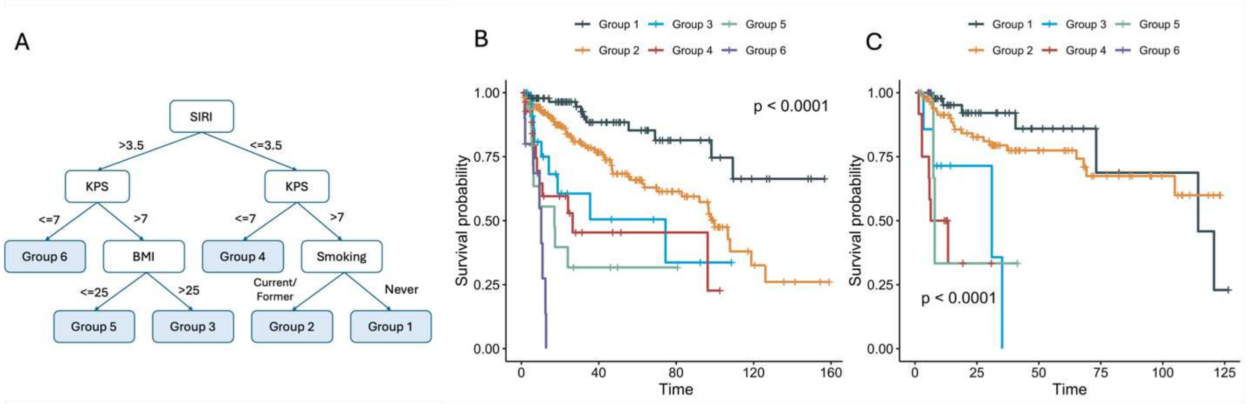

Supplemental Figure 2. (A) The decision tree with 4 variables stratifies the cohort into 6 groups. (B) Overall survival for the 6 risk groups clusters effectively as 3 groups. (C) The progression free survival curves of these 6 groups clustered effectively as 3 groups.
